# Reduced Left Ventricular Function on Cardiac MRI in SLE Patients Correlates with Measures of SLE Disease Activity and Inflammation

**DOI:** 10.26502/jrci.2809088

**Published:** 2023-12-29

**Authors:** Audrey M. Hagiwara, Erica Montano, Gantseg Tumurkhuu, Moumita Bose, Marianne Bernardo, Daniel S. Berman, Galen Cook Wiens, Michael D. Nelson, Daniel J. Wallace, Janet Wei, Mariko Ishimori, C. Noel Bairey Merz, Caroline Jefferies

**Affiliations:** 1 Division of Rheumatology, Department of Medicine, Cedars-Sinai Medical Center, Los Angeles, CA; 2 Kao Autoimmunity Institute, Cedars-Sinai Medical Center, Los Angeles, CA, USA; 3 S. Mark Taper Foundation Imaging Center, Cedars-Sinai Medical Center; 4 Department of Cardiology, Cedars-Sinai Medical Center; 5 Barbra Streisand Women’s Heart Center, Cedars-Sinai Heart Institute, Cedars-Sinai Medical Center; 6 Applied Physiology and Advanced Imaging Laboratory, University of Texas at Arlington, Texas, USA; 7 David Geffen School of Medicine at University of California Los Angeles (UCLA), Los Angeles, CA

**Keywords:** Coronary Microvascular Dysfunction, Myocardial Perfusion Reserve Index, Systemic lupus Erythematosus

## Abstract

**Background:**

Women with SLE have an elevated risk of CVD morbidity and mortality and frequently report chest pain in the absence of obstructive CAD. Echocardiographic studies often demonstrate reduced LV function, correlating with higher disease activity. We used cardiac MRI (cMRI) to investigate the relationship between SLE, related inflammatory biomarkers and cardiac function in female SLE patients.

**Methods:**

Women with SLE reporting chest pain with no obstructive CAD (n=13) and reference controls (n=22) were evaluated using stress-rest cMRI to measure LV structure, function, tissue characteristics, and myocardial perfusion reserve index (MPRI). Coronary microvascular dysfunction (CMD) was defined as MPRI <1.84. Serum samples were analyzed for inflammatory markers. Relationships between clinical and cMRI values of SLE subjects were assessed, and groups were compared.

**Results:**

40% of SLE subjects had MPRI < 1.84 on cMRI. Compared to controls, SLE subjects had higher LV volumes and mass and lower LV systolic function. SLICC DI was related to worse cardiac function and higher T1. CRP was related to higher cardiac output and a trend to better systolic function, while ESR and fasting insulin were related to lower LV mass. Lower fasting insulin levels correlated with increased ECV.

**Conclusions:**

Among our female SLE cohort, 40% had CMD, and SLICC DI correlated with worse cardiac function and diffuse fibrosis. Higher inflammatory markers and low insulin levels may associate with LV dysfunction. Our findings underline the potential of non-invasive cMRI as a tool for monitoring cardiovascular function in SLE patients.

## 1. Introduction

Systemic lupus erythematosus (SLE) is a sexually dimorphic autoimmune disease, with approximately 90% of patients being women. Chest pain is a common symptom in patients with SLE [1], with cardiovascular disease (CVD) being a significant contributor to morbidity and mortality in SLE. Compared to the general population, cardiovascular mortality is 2.7-fold higher in SLE patients [2]. The risk of CVD is significantly elevated in women with SLE, particularly in the 35 to 44 year age group who have a 50-fold increased risk of myocardial infarction (MI) compared to age-matched healthy individuals [3]. The origin of chest pain in SLE can be attributed to multiple causes, including pericardial disease, myocarditis, and coronary artery disease (CAD). However, coronary angiography is frequently normal, indicating causes other than CAD driving anginal chest pain [4].

With the advancement in non-invasive diagnostic imaging, it is now understood that many SLE patients have ischemia with no obstructive CAD (INOCA), which is associated with elevated risk for CVD events [5–8]. Studies using cardiac magnetic resonance imaging (cMRI) have demonstrated a high prevalence of INOCA amongst SLE patients, which is predominantly attributable to coronary microvascular dysfunction (CMD) [9–11]. Now a routine tool within cardiac diagnostics, cMRI measures determines the mass and volumes of the heart, in addition to providing structural imaging of the myocardial tissue to detect fibrosis. Stress perfusion imaging allows measurement of coronary blood flow, which permits detection of ischemia and valve disorders. The semi-quantitative myocardial perfusion index (MPRI) is both sensitive and specific for the diagnosis of CMD (a MPRI of less than 1.84 being diagnostic of CMD), whereas measures of left ventricular (LV) diastolic volumes and ejection fraction (EF) detect cardiac dysfunction [12]. CMRI MPRI is an independent predictor of major adverse cardiovascular events (MACE) in patients with INOCA [13]. In addition to cardiac positron emission tomography (PET), which is the most studied non-invasive method for diagnosing CMD, stress cMRI is a reliable tool for diagnosing CMD while also offering insight into cardiac anatomy and function [12, 14–16].

The pathogenic mechanisms underlying CVD in SLE are incompletely understood; however, inflammation, endothelial dysfunction, and autoimmunity are known contributing factors. For example, systemic inflammation results in the upregulation of endothelial adhesion molecules, increased recruitment of mononuclear cells, and the production of pro-inflammatory cytokines which propagate atherogenesis [17, 18]. Clinical measurements of inflammation in SLE include complement, erythrocyte sedimentation rate (ESR), and C-reactive protein (CRP), which are used in scoring tools to determine and follow SLE disease activity and organ damage. Activation of the complement system leads to low levels of C3 and C4 in SLE [19]. ESR and CRP are non-specific markers of inflammation that are elevated in SLE and are associated with organ inflammation and infection, respectively [20]. It is unknown whether markers of clinical inflammation in SLE correlate with cardiac function abnormalities on cMRI. As such, this study aims to correlate changes in cardiac function in SLE patients using advanced cardiac imaging with clinical, laboratory and disease activity profiles of the patients in order to better understand subclinical heart disease in SLE.

## 2. Patients and Methods

### 2.1 Patients and Methods

This cross-sectional study was approved by the institutional review board at Cedars-Sinai Medical Center, and all participants gave informed consent prior to participation. Participants were recruited from the rheumatologic clinic. Inclusion criteria consisted of female SLE subjects (aged 37 to 57 years at baseline) with chest pain due to suspected angina. Exclusion criteria included documented obstructive CAD and contraindications to coronary computed tomography angiography (CCTA) or cardiac magnetic resonance imaging (cMRI).

### 2.2 Clinical Evaluation

Demographic data of the patients were collected including age and smoking history. Participants completed standardized angina symptom questionnaires and quality of life measures related to angina, in addition to a demographic questionnaire [21, 22]. Clinical data was collected including vital signs and body mass index. SLE disease activity was collected using the SLE disease activity index (SLEDAI) [23] and the systemic lupus international collaborating clinics (SLICC) Damage Index (DI) [24]. In addition, serum samples were taken and evaluated for complete blood count, creatinine, protein, fasting blood sugar, fasting insulin, inflammatory markers (erythrocyte sedimentation rate (ESR), C-reactive protein (CRP), Complements (C3, C4), and antibodies (anti-antinuclear antibody (ANA), anti-double stranded DNA (dsDNA), ribonucleoprotein (RNP), anti-Smith (Sm), SSA (Ro), SSB (La), and anti-topoisomerase I (Scl). Obstructive CAD was excluded by noninvasive CCTA.

### 2.3 Cardiac Magnetic Resonance Imaging

Using a standardized cardiac magnetic resonance imaging (cMRI) protocol [25], participants underwent stress cMRI at 3.0-Tesla (Siemens Vida or Biograph, Erlangen Germany) using ECG-gating/phased array coil, with 0.05 mmol/kg gadolinium first-pass perfusion three slice stress (adenosine 140 μg/kg-1/min-1 or regadenoson 0.4 mg) followed by rest, function, myocardial characterization (T1 and T2 mapping), and late gadolinium enhancement. T1 mapping was acquired using the modified Look-Locker inversion recovery (MOLLI) method. Caffeine was withdrawn for 24 hours prior to stress testing. Beta blocker, calcium channel blocker and nitrate medications were with-held for 24-48 hours prior to stress testing, at physician discretion. Blood was collected for hematocrit measurement on the day of imaging for calculating extracellular volume (ECV) fraction.

### 2.4 Cardiac MRI Analysis

cMRI studies were analyzed for LV function, strain, volumes, cardiac output, mass, T1 and T2 mapping, using CVI42 software (Circle Cardiovascular Imaging Inc, Calgary, AB, Canada), by the Cedars-Sinai Medical Center cMRI Core Laboratory. Myocardial perfusion reserve index (MPRI) was calculated as previously published [12]. LV mass, end-systolic and end-diastolic volumes were indexed to the body surface area (BSA). Native T1, ECV, and T2 times were measured in the midseptal region of the midventricular slice. Abnormal cMRI demonstrating coronary microvascular dysfunction was defined as a MPRI less than 1.84.

### 2.5 Statistical Analysis

Measurements are expressed as a mean and standard deviation, median and range for continuous variables, and counts and percentages for categorical variables. Groups were compared using t tests for SLE vs RC, with Welch’s correction for unequal variances. Wilcoxon rank-sum test was used to analyze clinical and cardiac function data between patients with CMD and those with no CMD. One way ANOVA was used to compare among CMD, no CMD and HC. When significant, a Bonferroni correction was used for post hoc pair-wise tests. Spearman correlations were used to assess association between CMRI features and lab results. Effect size was calculated as Cohen’s *d* with 95% confidence intervals for comparing between CMD and no CMD on CMRI features. A significance level of 0.05 was used for all hypothesis tests.

## 3. Results

Female SLE patient baseline characteristics are summarized in Table 1. The average age of the patients (n=13) recruited was 45, with SLEDAI scores ranging from inactive (0-3) to moderately active (4-6). Vital signs and blood count were all normal and clinical labs were consistent with a diagnosis of SLE, with serum creatinine and CRP being elevated compared to reference controls, whilst complement proteins C3 and C4 were reduced. Interestingly, while systolic blood pressure, body mass index (BMI) and heart rate were all similar between reference controls and SLE patients, diastolic blood pressure (DBP) was raised in SLE patients compared to reference controls (78.4+9.3 vs. 59±11 mmHg, respectively).

CMRI results are shown in Table 2, comparing cardiac function of SLE patients with a reference control group from the same center (n=22, all female). Of note, the age range of patients and reference controls was similar (45±5 vs. 49±6 years, respectively). LV ejection fraction (LVEF) was significantly decreased in SLE patients compared to reference controls (59±7% compared to 64±5%, p=0.0291). LV mass index (LVMi) was also significantly increased compared to reference controls (47.31±9.3 ml/m^2^ compared to 41.34.5±4.3 ml/m^2^, p=0.0461). In addition, radial, circumferential and longitudinal strain were reduced significantly in SLE patients compared to reference controls. Of the SLE patients, none had evidence of segmental perfusion defects, consistent with absence of obstructive CAD. One subject had LVEF < 50% with normal perfusion and no late gadolinium enhancement, consistent with nonischemic cardiomyopathy. An additional 2/13 (15%) had evidence of late gadolinium enhancement; one with extensive patchy scar in the absence of elevated T2 and normal wall motion, consistent with prior myocarditis, and another with small basal midmyocardial scar of uncertain significance. Mean T2 relaxation time was 43±4 ms (range 36-50 ms).

**Figure 1:**
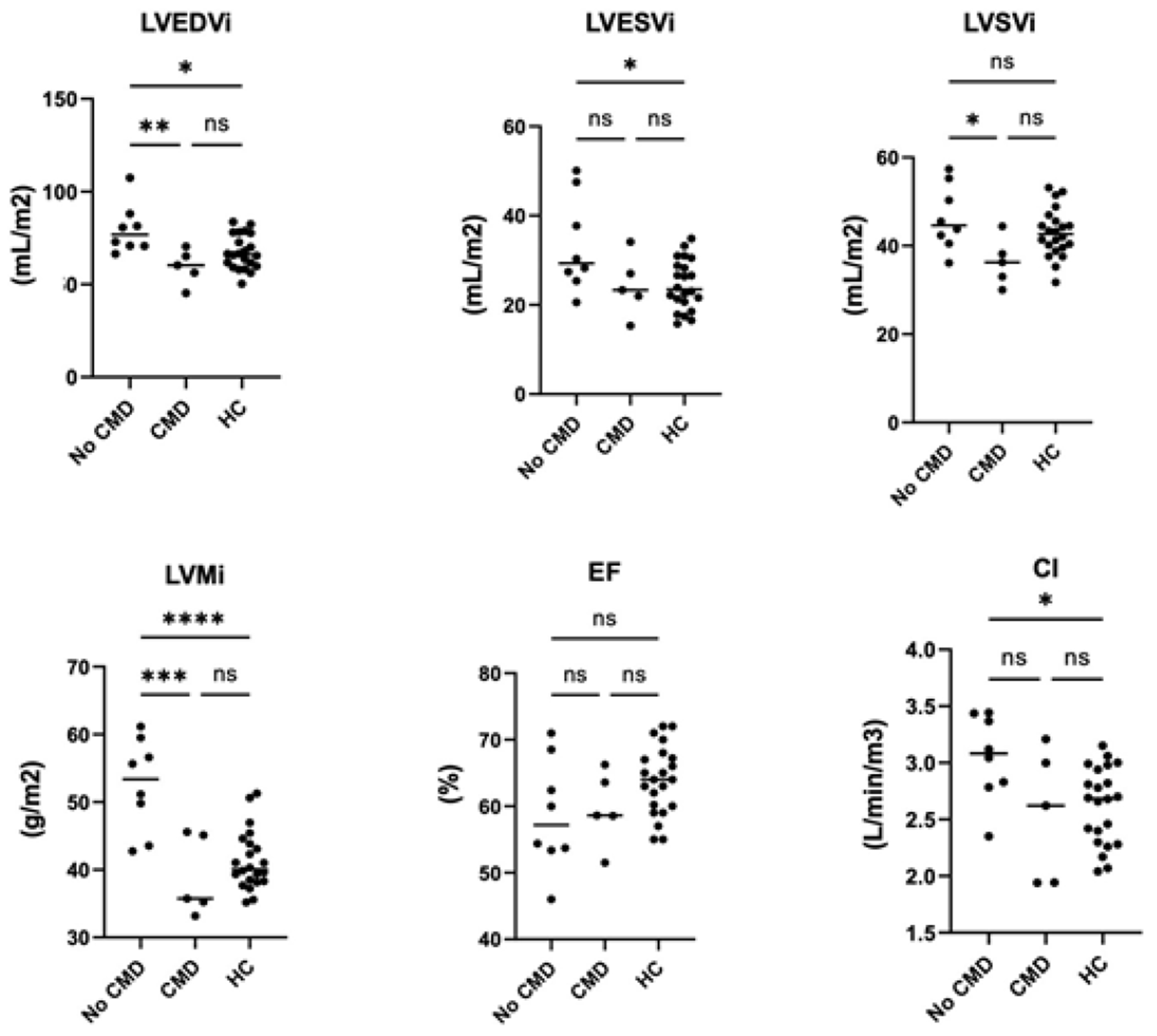
Comparison of cardiac structure and function in SLE patients with CMD or no CMD compared to reference controls. Statistical analysis was performed using One-Way ANOVA and Tukey’s multiple comparison test. ****p<0.0001, ***p<0.001, **p<0.01, *p<0.05, ns = not significant. LVEDVi: left ventricular end-diastolic volume index; LVESVi: left ventricular end-systolic volume index; LVSVi: left ventricular systolic volume index; LVMi: left ventricular mass index; EF: ejection fraction; CI: cardiac index

**Figure 2:**
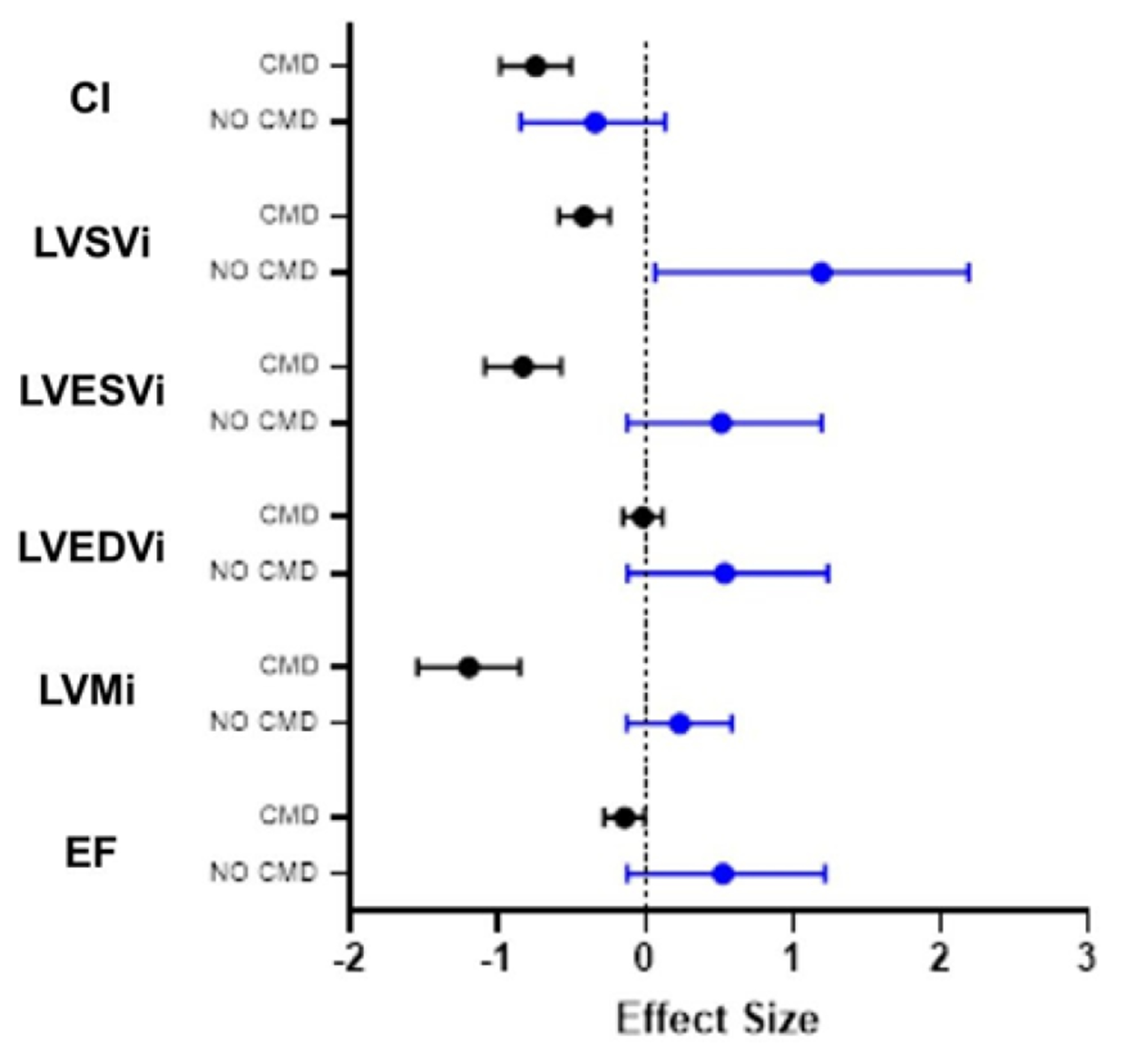
Patients without CMD (no CMD) show a stronger contribution to differences in cardiac structure and function between healthy controls and SLE patients. Patients with no CMD (blue) on MPRI show a stronger contribution to overall effect of SLE on cardiac function than do patients with CMD (black bars) when compared with reference controls. Data is represented as Cohen’s d + C.I. CI: cardiac index; LVSVi: left ventricular systolic volume index; LVESVi: left ventricular end-systolic volume index; LVEDVi: left ventricular end-diastolic volume index; LVMi: left ventricular mass index; EF: ejection fraction.

A Spearman correlation matrix was constructed to determine potential relationships between cardiac function measurements and clinical laboratory findings. Associations are shown in Table 3, with positive associations in green and negative associations in orange. We observed that the SLICC disease index (DI) score, which measures accumulated organ damage since onset of SLE, negatively correlated with LVEF, measures of strain but positively correlated with native T1 times. Fasting insulin levels and ESR showed a strong negative correlation with LVMi (Spearman r = −0.664 and −0.604, respectively) and fasting insulin levels also negatively correlated with ECV (r = −0.664). CRP, a marker of inflammation, showed a moderate-strong positive association with LVSVi and cardiac index and a similar trend towards a moderate negative association with longitudinal strain. We next analyzed the data based on whether SLE patients had CMD. Similar to previous work, we found that 5/13 (40%) of SLE subjects had CMD. No differences were observed between SLE patients with or without CMD when vital signs or clinical labs were compared (Table 1). When SLE cardiac function was assessed using Wilcoxon rank-sum test, we observed that LV function was significantly different between patients with CMD or no CMD (Table 4). However, no difference in EF or myocardial strain was observed between patients with or without CMD. We noted that patients without CMD had higher LVMi compared with either patients with CMD or reference controls, prompting us to assess the relative contribution of CMD versus no CMD to the overall differences in cardiac function between reference controls and SLE patients – ie EF, myocardial strain and LVMi. As shown in Figure 1, we observe that cardiac function was more similar between patients with CMD and reference controls, indicating that patients without CMD had a larger degree of contribution to the overall effect size noted previously. Figure 2 demonstrates how the patient group without CMD contributed to the overall differences observed. Overall, SLE patients without CMD (blue) contributed stronger to the effect seen between reference control cardiac MRI function than did SLE patients with CMD (black).

## 4. Discussion

A well-known consequence of systemic inflammation, as seen in SLE, are vascular changes, which contribute to the increased risk of CVD in SLE patients and alterations in cardiac function. Previous echocardiographic studies have reported alterations in LV function, increase in LVM, and LV hypertrophy in patients with SLE, in part associated with traditional risk factors of age, obesity, hypertension, and diabetes [26]. Our study is in agreement with these studies, with our cohort demonstrating increased LVMi and moreover, decreased LVEF and decreased myocardial strain, reflecting global LV remodeling contributing to LV dysfunction in our SLE cohort [26–29]. Alterations in myocardial strain in SLE patients, seen in our cohort as well as other studies, further reinforces the impact of SLE on cardiac function. Impairment in longitudinal strain has been suggested as an index for evaluating ventricular function and as a predictor of cardiovascular morbidity [30, 31]. Recent studies using speckle tracking echocardiography (STE) have reported that SLE patients show impairment in cardiac strain reflecting cardiac dysfunction [32–34]. Nikdoust et al. reported that SLE patients with no reported cardiac symptoms had significant decreases in longitudinal strain [32], whilst Taha et al. showed that SLE patients with active disease had impairment in longitudinal, circumferential and radial strain on STE [33]. In line with these studies, we have observed impaired cardiac strain on cMRI, which is comparable to STE in measuring strain, radial strain is decreased by approximately 20% in our SLE cohort compared to reference controls from the same center, and circumferential and longitudinal strain approximately 10% changed.

Interestingly, disease activity may also be associated to cardiac dysfunction in SLE patients. Both ESR and CRP are non-specific markers of inflammation, being increased in malignancy, infection and autoimmune disease [35]. In SLE, both markers tend to increase with disease exacerbations, making them potentially useful biomarkers of disease activity [20]. CRP levels have previously been associated with cardiac abnormalities in SLE patients on cMRI [6, 36]. Using STE, Zhong et al. demonstrated that disease activity and markers of inflammation (specifically CRP, ESR and C3/C4) correlated with decreased speckle strain parameters, indicating subclinical myocardial injury [34]. In our cohort, cardiac strain and LV function (EF, LVSV, and LVESV) show a moderate to strong correlation with SLICC DI, as a measure of damage accrual in SLE, in addition to markers of systemic inflammation and disease activity such as CRP and ESR. In addition, SLICC DI and CRP levels also correlate with changes in native T1 time and ECV, both of which are indicative of cardiac fibrosis, a strong predictor of subclinical LV dysfunction. Although, previous studies have shown that higher SLICC DI scores have been associated with macrovascular effects, with studies demonstrating SLE subjects with higher SLICC DI scores to have higher risk of developing MACE [37] and increased plaque development [38, 39], ours is the first study to demonstrate that SLICC DI may also be a useful tool in identifying microvascular effects and subclinical atherosclerotic disease.

In SLE, the systemic inflammation and release of proinflammatory cytokines negatively affect insulin signaling and result in insulin resistance [42, 43]. As such, fasting insulin levels are typically elevated in SLE patients [44]. Interestingly, our study demonstrated that lower fasting insulin levels were moderately associated with both increased LVM and ECV, with the latter associated with cardiac fibrosis. Insulin is known to play a role in adaption to myocardial stress, with insulin resistance contributing to worsening cardiac function [45]. Recent studies have identified low fasting insulin levels as an independent predictor of all-cause mortality and cardiovascular mortality in patients with acute decompensated heart failure without diabetes mellitus [46]. Insulin also affects fatty acid lipolysis, leading to reduced plasma fatty acid levels likely contributing to decreased effect of traditional CVD risk factors [47]. As such, our study suggests that low insulin levels in SLE patients may be an important prognostic factor in cardiac dysfunction.

In addition to macrovascular disease, SLE patients are at risk for microvascular dysfunction, as seen in our study which demonstrated that 40 % of SLE subjects had CMD on cMRI. Although the SLE subjects with CMD were overall similar to the subjects without CMD, subjects without CMD had higher LVMi compared to either SLE subjects with CMD or reference controls. This may have been driven by two SLE subjects who had MPRI > 1.84 and the highest LVMi and LVEDV of the group, possibly representing subclinical nonischemic SLE-related dilated cardiomyopathy phenotype. Since late gadolinium enhancement and myocardial T2 were normal in these two subjects, SLE myocarditis is an unlikely pathophysiologic mechanism for this finding. While LVMi is predictive of adverse cardiovascular events including heart failure [48–50], a larger sample size is needed to determine whether CMD in SLE patients with chest pain is related to lower LVMi or whether the absence of CMD suggests an alternative chest pain pathophysiology such as coronary vasospasm that may be related to higher LVMi. Since cMRI can identify early pathophysiologic structural and functional changes prior to the onset of clinically overt cardiovascular disease, cMRI has potential to enhance the understanding of SLE disease activity [51].

### 4.1 Limitations

This study has several limitations that should be addressed. First, this was a single center study with a small sample size. Future prospective studies with larger populations are required. Secondly, invasive coronary function testing was not carried out to diagnose coronary vasospasm to explain the patients without CMD but with chest pain had an alternative chest pathophysiology. Further research is required to determine this. Thirdly, further research is required to understand the contribution low insulin levels play in cardiac dysfunction in SLE, such as molecular assessment of immune cell responses to insulin and whether insulin signaling contributes to reduction of inflammation in SLE immune cells.

## 5. Conclusion

This study confirms the association between markers of inflammation in autoimmune disease such as ESR and CRP potential subclinical atherosclerotic disease in SLE patients on cMRI. It also reveals for the first time that patients with high SLICC DI may be more at risk for developing changes in left ventricular function and subclinical CVD. Similarly, low insulin levels were observed to correlate with increased LVMi and ECV, suggesting a cardioprotective effect of insulin in SLE patients. Our findings underline the potential of non-invasive cMRI as a tool for monitoring cardiovascular function in SLE, particularly in patients with high SLICC DI, ESR and CRP and low fasting insulin levels.

## Figures and Tables

**Table 1:** Baseline SLE Subject Characteristics overall and grouped into patients with CMD (CMD) or without CMD (no CMD). Results are presented as mean ± SD or median (min-max). Normal reference ranges are given in column 2 in square brackets. CMD and no CMD data was analyzed by unpaired student’s *t* test, with Welch’s correction as appropriate. In all cases, no significant difference between the two groups was observed.

Mean ±SD (range)	ALL SLE (n=13)	CMD (n=5)	No CMD (n=8)
**Age**	45 ± 5 years	43 (37-51) years	48 (43-57) years
**Ethnicity**			
Hispanic	3		
Non-Hispanic	10		
**SLEDAI**	2.5 ± 2.5	2.8 ± 3 (0-6)	1.4 ± 2 (0-6)
**SLICC**	---	0.8 ± 0.8 (0-2)	0.4 ± 1 (0-3)
**Clinical Labs**			
Fasting Blood Sugar	77.7 ± 7.9 mg/dl [100-125 mg/dl]	80.2 ± 6.5 mg/dl (72-90)	75.5 ± 10.1 mg/dl (65-91)
Fasting Insulin	9.6 ± 13.4mIU/ml [2-20 mIU/ml]	14.8 ± 15.4 mIU/ml (2-42)	4.5 ± 2.5 mIU/ml (1.4-7.6)
Serum Creatinine	1.26 ± 2.2 mg/dL [0.59-1.04 mg/dL]	0.67 ± 0.12 mg/dL (0.55-0.84)	0.72 ± 0.03 mg/dl (0.66-0.76)
Serum Protein	7.17 ± 1.04 g/dL [6-8 g/dL]	7.58 ± 1.47 g/dL (6.4-10.1)	7.09 ± 0.40 g/dl (6.8-7.9)
C3	93.5 ± 4.5 mg/dL [80-160 mg/dL]	124 ± 34 mg/dL (81-153)	108 ± 26 mg/dl (77-156)
C4	21.75 ± 7.5 mg/dL [15-45 mg/dL]	20 ± 11 mg/dL (10-32)	29 ± 12 mg/dl (22-51)
ESR	16.8 ± 20.38 mm/hr [0-29mm/hr]	32 ± 24 mm/hr (16-73)	14 ± 16 mm/hr (1-47)
CRP	1.84 ± 1.85 mg/dL [<1mg/dL]	2.6 ± 2.2 mg/dL (0.4-5.4)	3.6 ± 6.0 mg/dl (0.2-17.9)
Anti-ANA	---	424 ± 299 (50-640)	440 ± 277 (80-640)
Anti-DNA	---	<10	<10
RNP Antibody	---	68 ± 70.4 (2-156)	26.3 ± 46.8 (2-133)
Anti-Sm	---	17.2 ± 20.6 (2-52)	11.9 ± 18.6 (1-51)
SSA (Ro)	---	42.8 ± 55 (2-104)	10.4 ± 19.2 (1-56)
SSB (La)	---	22.8 ± 45.5(2-104)	5.5 ± 8.3 (1-25)
Anti-SCL	---	4.2 ± 3.3 (2-10)	1 ± 25 (1-25)
**Vital Signs**			
Systolic (mmHg)	115.8 ± 13.4	116.8 ± 7.4 (109-128)	119.4 ± 14 (104-127)
Diastolic (mmHg)	76.8 ± 11.3	78 ± 8.7 (70-92)	78.6 ± 10 (66-87)
Pulse (bpm)	69.8 ± 16.8 bpm	79 ± 13 (72-90)	65 ± 16 (49-89)
BMI (kg/m^2^)	25.3 ± 5.3	24.2 ± 4.9 (18-28)	27.5 ± 5.2 (19-33)
**Blood count**			
Hemoglobin	12.6 ± 1.3 g/dL [11.6-15 g/dL]	13.0 ± 1.6 g/dL (11.2-14.9)	12.6 ± 1.0 g/dL (11.1-13.9)
WBC	4.6 ± 1.5 109/L [4-10 109/L]	5.0 ± 1.37 109/L (3.4-6.8)	4.4 ± 1.58 109/L (3.1-7.2)
Platelets	250 ± 41 10^9^/L [157-371 10^9^/L]	256 ± 41 10^9^/L (157-371)	224 ± 46 10^9^/L (173-305)

**Table 2:** Comparison of cardiac structure and function between SLE patients and reference controls (RC) based on cMRI values.

Variable	SLE (n=13)	RC (n=22)	*p*-value
EF (%)	59 ± 7	64 ± 5	0.0291
LVM/LVEDV (g/mL)	0.66 ± 0.08	0.62 ± 0.07	ns
LVEDVi (mL/m^2^)	72.02 ± 15.4	67.40 ± 9.3	ns
LVESVi (mL/m^2^)	29.9 ± 10.2	24.5 ± 5.6	ns
LVSVi (mL/m^2^)	42.56 ± 8.2	42.92 ± 5.4	ns
LVMi (g/m^2^)	47.31 ± 9.3	41.34 ± 4.3	0.0454
CI (L/min/m^2^)	3 ± 1	2.6 ± 0.3	ns
Radial Strain (%)	29.70 ± 6.2	34.37 ± 5.8	0.0306
Circumferential Strain (%)	−17.94 ± 2.5	−19.73 ± 2	0.0251
Longitudinal Strain (%)	−18.40 ± 2.2	−20.10 ± 1.9	0.0202
Native T1 (ms)	1262.5 ± 34.6	1258 ± 47	ns
ECV (%)	28.7 ± 3.1	29.4 ± 2.6	ns

Results are shown as mean ± S.D. and significance was determined using student *t* test with or without Welch’s correction as appropriate. EF: ejection fraction, LVM: left ventricular mass; LVEDV: left ventricular end diastolic volume; BSA: body surface area; LVEDVi: left ventricular end diastolic volume index (LVEDV/BSA); LVESVi: left ventricular end systolic volume index (LVESV/BSA); LVSVi: left ventricular systolic volume index (LVSV/BSA); LVMi: left ventricular mass index (LVM/BSA); CI: cardiac index; ECV: extracellular volume. ns represents a p-value > 0.05.

**Table 3:** Analysis of cardiac structure and function versus clinical values from patients in the study.

		SLICC	CRP	Fasting Insulin	ESR
EF (%)	r	−0.545			
	p	0.057			
LVSVi (mL/m^2^)	r		0.528		
	p		0.066		
CO/BSA (L/min/m^2^)	r		0.566		
	p		0.047		
LVMi (g/m^2^)	r			−0.664	−0.604
	p			0.031	0.032
Radial Strain (%)	r	−0.743			
	p	0.005			
Circumferential Strain (%)	r	0.669			
	p	0.014			
Long strain (%)	r	0.501	0.528		
	p	0.083	0.066		
Native T1 (ms)	r	0.621			
	p	0.035			
T2 (ms)	r			0.701	
	p			0.02	
ECV (%)	r			−0.664	
	p			0.031	

A correlation matrix was constructed to determine potential relationships between cMRI values and clinical laboratory findings. Associations are shown and are represented as positive associations in the green boxes and negative associations in the orange boxes. The Spearman r coefficient and p value is shown in each case. EF: ejection fraction; LVSVi: left ventricular systolic volume index; CI: cardiac index; LVMi: left ventricular mass index; ECV: extracellular volume.

**Table 4:** Comparison of cardiac structure and function in SLE patients with CMD compared to SLE patients without on cMRI.

	CMD (N=5)	No CMD (N=8)	Total (N=13)	p value
**LVEDVi(mL/m^2^)**				0.0068
Median	60.3	76.8	70.7	
Q1, Q3	56.4, 65.2	70.8, 84.8	65.2, 80.7	
**LVESVi(mL/m^2^)**				ns
Median	23.3	29.3	27.4	
Q1, Q3	22.0, 27.1	26.4, 42.6	23.3, 34.1	
**LVMi(g/m^2^)**				0.0233
Median	35.7	53.4	45.6	
Q1, Q3	35.3, 45.1	46.7, 58.1	42.8, 55.6	
**LVSVi(mL/m^2^)**				0.0481
Median	36.3	44.6	42.4	
Q1, Q3	33.0, 38.2	41.5, 52.8	36.3, 45.5	
**LVEF(%)**				ns
Median	58.6	57.2	58.6	
Q1, Q3	58.5, 63.6	53.6, 63.9	53.7, 63.6	
**LVSV(mL)**				0.0451
Median	65.9	86.9	67.6	
Q1, Q3	51.9, 67.2	74.8, 95.2	64.3, 87.6	
**Radial Strain(%)**				ns
Median	27	27.7	27.5	
Q1, Q3	26.5, 35.7	27.0, 36.0	26.5, 36.0	
**Longitudinal Strain(%)**				ns
Median	−17.6	−19.1	−18.8	
Q1, Q3	−19.3, −16.8	−20.0, −17.9	−19.5, −16.8	
**Circumferential Strain(%)**				ns
Median	−17.2	−17.2	−17.2	
Q1, Q3	−20.3, −16.9	−20.4, −17.0	−20.4, −16.9	
**T1 (ms)**				ns
Median	1258	1261	1261	
Q1, Q3	1,25,01,278	1252.5, 1265.5	1245, 1272	
**T2(ms)**				ns
Median	44	42.5	43	
Q1, Q3	40, 45	40.8, 44.5	40, 45	
**ECV(%)**				ns
Median	29	28	28.5	
Q1, Q3	26, 29	27.5, 29.5	27, 29.3	

Wilcoxon rank-sum test was used to determine potential differences in cardiac function between patients with CMD (N=5) versus those without CMD (N=8). LVEDVi: left ventricular end-diastolic volume index; LVESVi: left ventricular end-systolic volume index; LVMi: left ventricular mass index, LVSV: left ventricular systolic volume; LVSVi: LVSV index; LVEF: left ventricular ejection fraction; ECV: extracellular volume.

## References

[1] Ishimori ML, Michael H, . Microvascular angina: an underappreciated cause of SLE chest pain. J Rheumato 40 (2013): 746–747. 10.3899/jrheum.121277 23637384

[2] Yurkovich M, Kateryna V, Wenjia C, . Overall and cause-specific mortality in patients with systemic lupus erythematosus: a meta-analysis of observational studies. Arthritis Care Res (Hoboken) 66 (2014): 608–616. 24106157 10.1002/acr.22173

[3] Manzi S, Meilahn EN, Rairie JE, . Age-specific incidence rates of myocardial infarction and angina in women with systemic lupus erythematosus: comparison with the Framingham Study. Am J Epidemiol 145 (1997): 408–415. 9048514 10.1093/oxfordjournals.aje.a009122

[4] Ishimori ML, Gal NJ, Rogatko A, . Prevalence of angina in patients with systemic lupus erythematosus. Arthritis Res Ther 14 (2012): A62.

[5] Manchanda AS, Kwan AC, Ishimori M, . Coronary Microvascular Dysfunction in Patients With Systemic Lupus Erythematosus and Chest Pain. Front Cardiovasc Med 9 (2022): 867155. 35498009 10.3389/fcvm.2022.867155 PMC9053571

[6] Sandhu VK, Janet W, Louise EJ, . Five-Year Follow-Up of Coronary Microvascular Dysfunction and Coronary Artery Disease in Systemic Lupus Erythematosus: Results from a Community-Based Lupus Cohort. Arthritis Care Res (Hoboken) 72 (2020): 882–887. 31058466 10.1002/acr.23920 PMC6832763

[7] Bairey MCN, Puja KM, Jingwen H, . Ischemia and No Obstructive Coronary Artery Disease (INOCA): Developing Evidence-Based Therapies and Research Agenda for the Next Decade. Circulation 135 (2017): 1075–1092. 28289007 10.1161/CIRCULATIONAHA.116.024534 PMC5385930

[8] Weber BN, Emma S, Leanne B, . Coronary Microvascular Dysfunction in Systemic Lupus Erythematosus. J Am Heart Assoc 10 (2021): 018555. 10.1161/JAHA.120.018555 PMC8403317 34132099

[9] Kiani AN, Laurence SM, Wendy SP, . Coronary calcification in SLE: comparison with the Multi-Ethnic Study of Atherosclerosis. Rheumatology (Oxford) 54 (2015): 1976–1981. 26106213 10.1093/rheumatology/kev198 PMC4715250

[10] Gartshteyn Y, Genna B, Sharan M, . Prevalence of coronary artery calcification in young patients with SLE of predominantly Hispanic and African-American descent. Lupus Sci Med 6 (2019): e000330. 31321063 10.1136/lupus-2019-000330 PMC6606070

[11] Ishimori ML, Rebecca M, Daniel SB, . Myocardial ischemia in the absence of obstructive coronary artery disease in systemic lupus erythematosus. JACC Cardiovasc Imaging 4 (2011): 27–33. 21232700 10.1016/j.jcmg.2010.09.019

[12] Thomson LE, Janet W, Megha A, . Cardiac magnetic resonance myocardial perfusion reserve index is reduced in women with coronary microvascular dysfunction. A National Heart, Lung, and Blood Institute-sponsored study from the Women’s Ischemia Syndrome Evaluation. Circ Cardiovasc Imaging 8 (2015). 10.1161/CIRCIMAGING.114.002481 PMC4375783 25801710

[13] Zhou W, Jonan C, Siu TL, . Long-Term Prognosis of Patients With Coronary Microvascular Disease Using Stress Perfusion Cardiac Magnetic Resonance. JACC Cardiovasc Imaging 14 (2021): 602–611. 33248966 10.1016/j.jcmg.2020.09.034

[14] Zorach B, Peter WS, Jamieson B, . Quantitative cardiovascular magnetic resonance perfusion imaging identifies reduced flow reserve in microvascular coronary artery disease. J Cardiovasc Magn Reson 20 (2018): 14. 29471856 10.1186/s12968-018-0435-1 PMC5822618

[15] Kotecha T, Ana MN, Michele B, . Automated Pixel-Wise Quantitative Myocardial Perfusion Mapping by CMR to Detect Obstructive Coronary Artery Disease and Coronary Microvascular Dysfunction: Validation Against Invasive Coronary Physiology. JACC Cardiovasc Imaging 12 (2019): 1958–1969. 30772231 10.1016/j.jcmg.2018.12.022 PMC8414332

[16] Marano P, Wei J, Merz CNB. Coronary Microvascular Dysfunction: What Clinicians and Investigators Should Know. Curr Atheroscler Rep 25 (2023): 435–446. 37338666 10.1007/s11883-023-01116-z PMC10412671

[17] Bartoloni E, Shoenfeld Y, Gerli R. Inflammatory and autoimmune mechanisms in the induction of atherosclerotic damage in systemic rheumatic diseases: two faces of the same coin. Arthritis Care Res (Hoboken) 63 (2011): 178–183. 20740611 10.1002/acr.20322

[18] Prasad M, Joerg H, Sherine EG, . Cardiorheumatology: cardiac involvement in systemic rheumatic disease. Nat Rev Cardiol 12 (2015): 168–176. 25533796 10.1038/nrcardio.2014.206 PMC4641514

[19] Weinstein A, Alexander RV, Zack DJ. A Review of Complement Activation in SLE. Curr Rheumatol Rep 23 (2021): 16. 33569681 10.1007/s11926-021-00984-1 PMC7875837

[20] Aringer M. Inflammatory markers in systemic lupus erythematosus. J Autoimmun 110 (2020): 102374. 31812331 10.1016/j.jaut.2019.102374

[21] Rose GA, The diagnosis of ischaemic heart pain and intermittent claudication in field surveys. Bull World Health Organ 27 (1962): 645–658. 13974778 PMC2555832

[22] Rahman MA, Nicola S, Mohammad Afzal M, . Rose Angina Questionnaire: validation with cardiologists’ diagnoses to detect coronary heart disease in Bangladesh. Indian Heart J 65 (2013): 30–39. 23438610 10.1016/j.ihj.2012.09.008 PMC3861154

[23] Bombardier C, Gladman DD, Urowitz MB, . Derivation of the SLEDAI. A disease activity index for lupus patients. The Committee on Prognosis Studies in SLE. Arthritis Rheum 35 (1992): 630–640. 1599520 10.1002/art.1780350606

[24] Petri M, Ana MO, Graciela SA, . Derivation and validation of the Systemic Lupus International Collaborating Clinics classification criteria for systemic lupus erythematosus. Arthritis Rheum 64 (2012): 2677–2686. 22553077 10.1002/art.34473 PMC3409311

[25] Aldiwani H, Michael DN, Behzad S, . Reduced myocardial perfusion is common among subjects with ischemia and no obstructive coronary artery disease and heart failure with preserved ejection fraction: a report from the WISE-CVD continuation study. Vessel Plus 6 (2022). 10.20517/2574-1209.2021.103 PMC9278056 35836794

[26] Pieretti J, Mary JR, Richard BD, . Systemic lupus erythematosus predicts increased left ventricular mass. Circulation 116 (2007): 419–426. 17620509 10.1161/CIRCULATIONAHA.106.673319

[27] Gaudron P, Eilles C, Kugler I, . Progressive left ventricular dysfunction and remodeling after myocardial infarction. Potential mechanisms and early predictors. Circulation 87 (1992): 755–763. 10.1161/01.cir.87.3.755 8443896

[28] McGhie AI, Willerson JT, Corbett JR. Radionuclide assessment of ventricular function and risk stratification after myocardial infarction. Circulation 84 (1991): I167–1176. 1884482

[29] Winslow TM, Ossipov MA, Fazio GP, . The left ventricle in systemic lupus erythematosus: Initial observations and a five-year follow-up in a university medical center population. Am Heart J 125 (1993): 1117–1122. 8465737 10.1016/0002-8703(93)90123-q

[30] Potter E, Marwick TH. Assessment of Left Ventricular Function by Echocardiography: The Case for Routinely Adding Global Longitudinal Strain to Ejection Fraction. JACC Cardiovasc Imaging 11 (2018): 260–274. 29413646 10.1016/j.jcmg.2017.11.017

[31] Biering ST, Sofie RBS, Flemming JO, . Global Longitudinal Strain by Echocardiography Predicts Long-Term Risk of Cardiovascular Morbidity and Mortality in a Low-Risk General Population: The Copenhagen City Heart Study. Circ Cardiovasc Imaging 10 (2017). 10.1161/CIRCIMAGING.116.005521 PMC5363277 28264868

[32] Nikdoust F, Elham B, Seyed AT, . Early diagnosis of cardiac involvement in systemic lupus erythematosus via global longitudinal strain (GLS) by speckle tracking echocardiography. J Cardiovasc Thorac Res 10 (2018): 231–235. 30680083 10.15171/jcvtr.2018.40 PMC6335988

[33] Taha M, Dina L, Yasser B, . Subclinical left ventricular dysfunction during systemic lupus erythematosus activity with follow-up after remission – A speckle tracking echocardiographic study. The Egyptian Rheumatol 44 (2022): 279–285.

[34] Zhong X, Lixin C, Guijuan P, . Early assessment of subclinical myocardial injury in systemic lupus erythematosus by two-dimensional longitudinal layer speckle tracking imaging. Quant Imaging Med Surg 12 (2022): 2947–2960. 35502373 10.21037/qims-21-805 PMC9014157

[35] Bray C, Lauren NB, Hong L, . Erythrocyte Sedimentation Rate and C-reactive Protein Measurements and Their Relevance in Clinical Medicine. WMJ 115 (2016): 317–321. 29094869

[36] Braggion SMF, Mohamed AA, Evangelos G, . Myocardial late gadolinium enhancement in systemic lupus erythematosus as a marker of chronic inflammation. J Cardiovasc Magn Reson 15 (2013): 121.

[37] Haque S, Sarah S, CR . Progression of subclinical and clinical cardiovascular disease in a UK SLE cohort: the role of classic and SLE-related factors. Lupus Sci Med 5 (2018): 000267. 10.1136/lupus-2018-000267 PMC6257381 30538814

[38] Roman MJ, Beth AS, Adrienne D, . Prevalence and correlates of accelerated atherosclerosis in systemic lupus erythematosus. N Engl J Med 349 (2003): 2399–406. 14681505 10.1056/NEJMoa035471

[39] Roman MJ, Mary KC, Michael D, . Rate and determinants of progression of atherosclerosis in systemic lupus erythematosus. Arthritis Rheum 56 (2007): 3412–3419. 17907140 10.1002/art.22924

[40] Van Niekerk G, Claudia C, Daleen C, . Insulin as an immunomodulatory hormone. Cytokine Growth Factor Rev 52 (2020): 34–44. 31831339 10.1016/j.cytogfr.2019.11.006

[41] Jeschke MG, Darren FB, Celeste C, . Effect of insulin on the inflammatory and acute phase response after burn injury. Crit Care Med 35 (2007): 519–523. 17713402 10.1097/01.CCM.0000282027.10288.10

[42] Wang X, Wei B, Jun L, . Inflammatory markers and risk of type 2 diabetes: a systematic review and meta-analysis. Diabetes Care 36 (2013): 166–175. 23264288 10.2337/dc12-0702 PMC3526249

[43] Escarcega RO, Luis JJ, Jorge RR, . Insulin resistance, chronic inflammatory state and the link with systemic lupus erythematosus-related coronary disease. Autoimmun Rev 6 (2006): 48–53. 17110317 10.1016/j.autrev.2006.07.001

[44] Miyake CNH, Bruno G, Wagner SD, . Increased Insulin Resistance and Glucagon Levels in Mild/Inactive Systemic Lupus Erythematosus Patients Despite Normal Glucose Tolerance. Arthritis Care Res (Hoboken) 70 (2018): 114–124. 28320046 10.1002/acr.23237

[45] Rahman A, Syed J, Khursheed J, . Malnutrition and cachexia in heart failure. J Parenter Enteral Nutr 40 (2016): 475–486. 10.1177/0148607114566854 25634161

[46] Nogi M, Rika K, Satomi I, . Low Insulin Is an Independent Predictor of All-Cause and Cardiovascular Death in Acute Decompensated Heart Failure Patients Without Diabetes Mellitus. J Am Heart Assoc 9 (2020): e015393. 32406318 10.1161/JAHA.119.015393 PMC7660870

[47] Grossman AN, Lionel HO, Joni RB, . Glucose-insulin-potassium revived: current status in acute coronary syndromes and the energy-depleted heart. Circulation 127 (2013): 1040–1048. 23459576 10.1161/CIRCULATIONAHA.112.130625

[48] Tsao CW, Philimon NG, Carol JS, . Left Ventricular Structure and Risk of Cardiovascular Events: A Framingham Heart Study Cardiac Magnetic Resonance Study. J Am Heart Assoc 4 (2015): 002188. 10.1161/JAHA.115.002188 PMC4599505 26374295

[49] Bluemke DA, Richard AK, João ACL, . The relationship of left ventricular mass and geometry to incident cardiovascular events: the MESA (Multi-Ethnic Study of Atherosclerosis) study. J Am Coll Cardiol 52 (2008): 2148–2155. 19095132 10.1016/j.jacc.2008.09.014 PMC2706368

[50] De Simone G, John SG, Marcello C, . Left ventricular mass predicts heart failure not related to previous myocardial infarction: the Cardiovascular Health Study. Eur Heart J 29 (2008): 741–747. 18204091 10.1093/eurheartj/ehm605

[51] Mavrogeni S, Pepe A, Nijveldt R, . Cardiovascular magnetic resonance in autoimmune rheumatic diseases: a clinical consensus document by the European Association of Cardiovascular Imaging. Eur Heart J Cardiovasc Imaging 23 (2022): 308–322. 10.1093/ehjci/jeac134 35808990

